# Childhood sleep health and epigenetic age acceleration in late adolescence: Cross‐sectional and longitudinal analyses

**DOI:** 10.1111/apa.16719

**Published:** 2023-03-03

**Authors:** David Balfour, Phillip E. Melton, Joanne A. McVeigh, Rae‐Chi Huang, Peter R. Eastwood, Sian Wanstall, Amy C. Reynolds, Sarah Cohen‐Woods

**Affiliations:** ^1^ College of Education, Psychology, and Social Work Flinders University Adelaide South Australia Australia; ^2^ Menzies Institute for Medical Research, College of Health and Medicine University of Tasmania Hobart Tasmania Australia; ^3^ School of Population and Global Health The University of Western Australia Nedlands Western Australia Australia; ^4^ Curtin School of Allied Health Curtin University Perth Western Australia Australia; ^5^ Movement Physiology Laboratory, School of Physiology University of the Witwatersrand Johannesburg South Africa; ^6^ Nutrition and Health Innovation Research Institute (NHIRI) Edith Cowan University Perth Western Australia Australia; ^7^ Flinders Health and Medical Research Institute (Sleep Health) Flinders University Adelaide South Australia Australia; ^8^ Flinders University Institute for Mental Health and Wellbeing Flinders University Adelaide South Australia Australia; ^9^ Flinders Centre for Innovation in Cancer Flinders University Adelaide South Australia Australia

**Keywords:** biological age, childhood, DNA methylation, methylation age, the Raine Study

## Abstract

**Aim:**

Investigate if childhood measures of sleep health are associated with epigenetic age acceleration in late adolescence.

**Methods:**

Parent‐reported sleep trajectories from age 5 to 17, self‐reported sleep problems at age 17, and six measures of epigenetic age acceleration at age 17 were studied in 1192 young Australians from the Raine Study Gen2.

**Results:**

There was no evidence for a relationship between the parent‐reported sleep trajectories and epigenetic age acceleration (*p* ≥ 0.17). There was a positive cross‐sectional relationship between self‐reported sleep problem score and intrinsic epigenetic age acceleration at age 17 (*b* = 0.14, *p* = 0.04), which was attenuated after controlling for depressive symptom score at the same age (*b* = 0.08, *p* = 0.34). Follow‐up analyses suggested this finding may represent greater overtiredness and intrinsic epigenetic age acceleration in adolescents with higher depressive symptoms.

**Conclusion:**

There was no evidence for a relationship between self‐ or parent‐reported sleep health and epigenetic age acceleration in late adolescence after adjusting for depressive symptoms. Mental health should be considered as a potential confounding variable in future research on sleep and epigenetic age acceleration, particularly if subjective measures of sleep are used.

**Abbreviations**
CBCL: Child Behaviour Checklist for Ages 4–18
EAA: Epigenetic age acceleration
ExtrinsicAA: Extrinsic epigenetic age acceleration
GrimAA: AgeAccelGrim
HorvathAA: AgeAccelHorvath
IntrinsicAA: Intrinsic epigenetic age acceleration
BMI: Body mass index
PhenoAA: DNAm PhenoAgeAccel
SkinAA: AgeAccelSkinClock
YSR: Youth Self Report for Ages 11–18

**Key notes**

- Investigated if parent‐reported sleep trajectories from age 5 to 17 and self‐reported sleep problems at age 17 were associated with epigenetic age acceleration at age 17.
- No evidence for a relationship between self‐ or parent‐reported sleep health and epigenetic age acceleration after adjusting for depressive symptoms.
- Findings emphasise the need to consider mental health as a potential confounding variable in future research on sleep and epigenetic age acceleration.

## 1 INTRODUCTION

Poor sleep has been associated with poor physical and mental health, including increased risk of heart disease and type 2 diabetes, and higher symptoms of depression and anxiety.^1^ Healthy sleep is important in childhood and adolescence, as shown by associations with physical growth, mental health, brain maturation, and biomarkers of cardiometabolic risk.^2^, ^3^ One mechanism through which sleep may affect long‐term health is biological ageing.^4^ Specifically, poor sleep may affect cellular metabolism and disrupt or impair important restorative processes that occur during healthy sleep, leading to a cascade of deteriorative processes associated with advanced age.^4^

Biological ageing can be studied using epigenetic age acceleration (EAA), which is a representation of the extent to which epigenetic age—a molecular measure of biological age—deviates from calendar age. Higher EAA indicates higher than expected biological age based on calendar age, whereas negative EAA represents lower than expected biological age. EAA can be calculated using information about DNA methylation, which is a modification to the structure of DNA that can affect gene expression without altering the sequence of bases composing the genome.^5^ DNA methylation can occur at a cytosine‐phosphate‐guanine (CpG) site, where a guanine base follows as cytosine. A chemical known as a methyl group may be attached to the cytosine to form 5‐methylcytosine, potentially reducing the likelihood that the underlying genetic material will be expressed as a protein. Specific patterns of DNA methylation are associated with age in humans, so they can be used to represent biological age. Different algorithms, known as ‘epigenetic clocks’, can be used to calculate different types of EAA. The various types of EAA represent different aspects of biological age. Measures of EAA have been associated with several adverse health outcomes, including cancer, heart disease, type 2 diabetes, and all‐cause mortality,^5^, ^6^, ^7^ many of which have also been associated with poor sleep.^1^ These markers have also been associated with various environmental exposures, including stress, diet, and air pollution.^5^ EAA may therefore be a useful tool for identifying modifiable determinants of ageing and long‐term health in childhood and adolescence.

Several studies have reported evidence for a positive relationship between poor sleep and EAA in adults,^8^, ^9^, ^10^ with the largest study to date reporting higher EAA in middle‐aged and older adults with poorer self‐reported sleep.^11^ Evidence regarding the hypothesised relationship is limited in younger people, however. Only one previous study has investigated sleep and EAA in adolescence, with Carskadon et al.^12^ reporting increased EAA from baseline to 9‐week follow‐up in 18‐ and 19‐year‐old female college students with both short and irregular sleep, as measured by sleep diary. There was no evidence for a cross‐sectional relationship between sleep and EAA, but the study was conducted in a very small sample (*N* = 12), which may have impacted the findings. We aimed to supplement the emerging literature on sleep and EAA in adolescence by investigating if childhood measures of sleep health are associated with EAA in late adolescence. We studied six types of EAA, four of which had not been studied previously in relation to sleep in adolescents (ExtrinsicAA, IntrinsicAA, DNAm PhenoAgeAccel: PhenoAA, and AgeAccelSkinClock: SkinAA).

## 2 METHODS

### 2.1 Participants

We used data from the Raine Study, a prospective cohort study established in Western Australia in 1989. A total of 2868 children, referred to as Gen2, were born and recruited into the study from 1989 to 1992. Whole blood was collected at the 17 year follow‐up, providing 1192 samples for DNA methylation analysis. Apart from a minor bias toward higher socioeconomic status, Gen2 participants at the 17 year follow‐up are representative of the wider population in Western Australia (see Appendix S1). This study was approved by the University of Western Australia Human Research Ethics Committee (RA/4/20/5722) and the Flinders University Human Research Ethics Committee (HEL4639‐1).

### 2.2 Procedure

#### 2.2.1 Parent‐reported sleep trajectories from age 5 to 17

Sleep was assessed using the Child Behaviour Checklist for Ages 4–18 (CBCL),^13^ which was administered at the 5, 8, 10, 14, and 17 year follow‐ups. The CBCL includes 112 statements about child and adolescent behaviour, six of which are related to sleep: “nightmares,” “overtired without good reason,” “sleeps less than most kids,” “sleeps more than most kids during day and/or night,” “talks or walks in sleep”, and “trouble sleeping”. Parents were asked to indicate how much each statement applied to their child in the past 6 months: “not true (as far as you know)”, “somewhat or sometimes true”, or “very true or often true”. The individual items were scored from 0 to 2, and – following a widely‐used procedure – the scores were summed to produce a total sleep problem score from 0 to 12.^14^ In a previous study, McVeigh et al.^15^ used latent class growth analysis on the total scores to identify three trajectories, representing parent‐reported sleep health from age 5 to 17: consistently minimal sleep problems, moderate but declining sleep problems, and persistent sleep problems. The trajectories obtained by McVeigh et al.^16^ were used in the present study.

#### 2.2.2 Self‐reported sleep problems at age 17

Gen2 participants completed the Youth Self Report for Ages 11–18 (YSR)^13^ at the 17 year follow‐up. The YSR is a companion questionnaire to the CBCL, featuring a self‐report version of each sleep item on the parent‐report form except “talks or walks in sleep”. Scores were summed to yield a self‐reported sleep problem score from 0 to 10. Cohen's κ was calculated to represent agreement between nominally equivalent items on the YSR and the CBCL at age 17, to informally evaluate the level of response bias in the parent‐reported scores that were used to construct the trajectories (see Appendix S1).

#### 2.2.3 Blood, DNA methylation, and epigenetic age acceleration at age 17

Whole blood was collected at the 17 year follow‐up and blood DNA methylation was measured using the Illumina Infinium HumanMethylation450K BeadChip array. Data processing and quality control procedures were performed as described previously by Huang et al.^16^ We used Horvath's New Methylation Age Calculator^17^ to calculate six measures of EAA (see Appendix S1), described in Table 1. Following the approach used by Chen et al.,^6^ abundance estimates for seven white blood cell types were calculated from DNA methylation data and controlled for when computing IntrinsicAA (see Appendix S1).

**TABLE 1:** Measures of epigenetic age acceleration.

Measure	Method	Interpretation
AgeAccelGrim (GrimAA)	Predicts time‐to‐death from age, sex, and DNA methylation‐based estimates of smoking history and mortality‐related plasma proteins. Positive GrimAA represents lower predicted time‐to‐death than expected based on calendar age. Constructed using whole blood DNA methylation data	Captures epigenetic differences associated with smoking history and mortality‐related plasma proteins
AgeAccelHorvath (HorvathAA)	Predicts calendar age. Developed using DNA methylation data from 51 cell and tissue types, including whole blood	Captures epigenetic differences that are not specific to a particular cell or tissue type, having been constructed using DNA methylation data from a wide range of sample types^22^
Intrinsic epigenetic age acceleration (IntrinsicAA)	Equivalent to HorvathAA, but controls for DNA methylation‐based estimates of the abundance of white blood cell types that vary with calendar age. This is important because cell composition is a known confounder in DNA methylation research, as different cell types have different patterns of DNA methylation^6^	Captures age‐related differences that are not specific to a particular cell or tissue type, like HorvathAA.^17^ Controls for white blood cell composition, so may be more strongly weighted to measure aspects of age that vary within rather than between cells (i.e., cell‐intrinsic)^6^
Extrinsic epigenetic age acceleration (ExtrinsicAA)	Predicts calendar age from DNA methylation and weights the model to include information about DNA methylation‐based estimates of the abundance of white blood cell types that vary with calendar age. Developed using DNA methylation data from whole blood	Captures age‐related differences that are specific to whole blood. May be more strongly weighted to represent immune ageing^6^
AgeAccelSkinClock (SkinAA)	Predicts calendar age. Developed using DNA methylation data from buccal cells, cord blood, epithelial cells, fibroblasts, skin cells, and whole blood	Captures various cell‐intrinsic hallmarks of age, including deregulated nutrient sensing, mitochondrial dysfunction, stem cell exhaustion, and altered cell‐to‐cell communication^22^
DNAm PhenoAgeAccel (PhenoAA)	Predicts calendar age and nine biomarkers related to mortality risk, including immune, metabolic, and inflammatory markers. Constructed using DNA methylation data from whole blood	Captures epigenetic differences associated with various mortality‐related biomarkers in blood

### 2.3 Statistical analysis

Analyses were performed using Stata/MP 16.0. Analyses of variance were used to test for differences in EAA between the trajectories, and linear regression models were used to predict EAA from current sleep problems at age 17. Following the approach used by McVeigh et al.,^15^ we used the analytic weights function in Stata to weight the trajectory analyses by probability of trajectory membership. This approach weighted participants with a higher probability more strongly, reflecting greater certainty about the assigned trajectory. We constructed four models (outlined in Table 2), to estimate the independent effect of sleep on EAA while controlling for relevant covariates at age 17. Each covariate was selected because it has been separately associated with sleep and EAA in previous research (see Appendix S1).

**TABLE 2:** Covariates adjusted for in each model.

Model	Covariates at the 17 year follow‐up
Age‐adjusted	Calendar age
Demographic	Age‐adjusted + ethnicity (self‐reported), family income (parent‐reported), sex
Lifestyle	Demographic + BMI, smoking (self‐reported, past 4 weeks)
Fully‐adjusted	Lifestyle + depressive symptoms (Beck Depression Inventory for Youth), physical development (Tanner stage)

*Note*: All covariates were obtained at the 17 year follow‐up. See the Appendix S1 for more information.

## 3 RESULTS

### 3.1 Preliminary analyses of participant characteristics

Preliminary analyses of participant characteristics at age 17 and sleep trajectory from age 5 to 17 are reported in Table 3. Calendar age differed slightly between the persistent and minimal trajectories (*b* = 0.17, *p* = 0.01), suggesting older participants were more likely to have persistent parent‐reported sleep problems. Body mass index (BMI) also differed between the trajectories, with a lower mean in the minimal trajectory compared to the persistent trajectory (*b* = 1.60, *p* = 0.005). We also observed a difference in family income, with a lower mean in the persistent trajectory ($50 001 to $60 000 bracket) compared to the minimal trajectory ($60 001 to $70 000 bracket; *b* = −0.62, *p* = 0.004; Table S2). Depressive symptoms also differed, with a higher mean in the persistent trajectory and the declining trajectory than in the minimal trajectory (*b* = 5.92, *p* <0.0001; *b* = 2.75, *p* <0.0001), and a higher mean in the persistent trajectory than in the declining trajectory (*b* = 3.18, *p* = 0.04). Finally, self‐ and parent‐reported sleep problem scores were higher in the persistent trajectory than in the minimal trajectory (*b* = 1.91, *p* <0.0001; *b* = 3.35, *p* <0.0001). Additional preliminary analyses of participant characteristics, self‐reported sleep problems, and EAA at age 17 are reported in Table 4.

**TABLE 3:** Participant characteristics by sleep trajectory and among all participants assigned to a trajectory (*n* = 1053).

Characteristic	Consistently minimal sleep problems	Moderate but declining sleep problems	Persistent sleep problems	Total	Comparison between trajectories
Participants (*n*, %)	470 (44.63)	504 (47.86)	79 (7.50)	1053 (100)	
Sex (female; *n*, %)	231 (49.15)	246 (48.81)	42 (53.16)	519 (49.29)	χ^*2*^ = 0.52 (0.77)
**Age (*M*, *SD*)**	**17.20 (0.52)**	**17.18 (0.50)**	**17.39 (0.74)**	**17.21 (0.53)**	***F* = 4.49 (0.01)**
Age (range)	16.01–19.56	16.28–19.42	16.65–19.70	16.01–(19.70)	
Epigenetic age (*M*, *SD*)					
GrimEA	25.89 (3.07)	25.87 (3.24)	26.34 (2.74)	25.91 (3.13)	
HannumEEA	13.49 (5.58)	13.82 (5.64)	13.87 (5.57)	13.68 (5.61)	
HorvathEA	23.04 (3.48)	22.93 (3.83)	23.47 (3.55)	23.02 (3.66)	
PhenoEA	9.04 (5.78)	9.11 (6.15)	8.91 (5.18)	9.06 (5.91)	
SkinEA	19.04 (2.24)	19.00 (2.38)	19.13 (2.50)	19.03 (2.33)	
**BMI (*M*, *SD*)**	**22.49 (3.76)**	**23.57 (4.80)**	**24.30 (5.23)**	**23.14 (4.44)**	***F* = 7.33 (0.0007)**
Smoking (past 4 weeks; *n*, %)	70 (14.89)	92 (16.27)	14 (17.72)	176 (16.71)	χ^*2*^ = 2.46 (0.29)
Probability of sleep trajectory membership (*M*, *SD*)	0.87 (0.14)	0.87 (0.14)	0.86 (0.16)	0.87 (0.14)	*F* = 0.06 (0.94)
**Self‐reported sleep problems (*M*, *SD*)**	**2.12 (1.79)**	**2.71 (2.03)**	**3.94 (2.26)**	**2.53 (2.00)**	***F* = 27.08 (<0.0001)**
**Parent‐reported sleep problems (*M*, *SD*)**	**0.22 (0.57)**	**0.91 (1.21)**	**3.64 (2.23)**	**0.80 (1.39)**	***F* = 279.00 (<0.0001)**
**Depressive symptoms (*M, SD*)**	**6.96 (7.71)**	**9.45 (9.96)**	**12.51 (10.43)**	**8.53 (9.19)**	***F* = 11.90 (<0.0001)**
Ethnicity (*n*, %)					χ^*2*^ = 9.85 (0.28)
African or African American	25 (5.32)	18 (3.57)	5 (6.33)	48 (4.56)	
East Asian	71 (15.11)	63 (12.50)	8 (10.13)	142 (13.49)	
European	343 (72.98)	393 (77.98)	62 (78.48)	798 (75.78)	
South Asian	8 (1.70)	18 (3.57)	2 (2.53)	28 (2.66)	
Other	10 (2.13)	9 (1.79)	0 (0.00)	19 (1.80)	
**Family income (0 to 11; *M*, *SD*)**	**8.67 (2.75)**	**8.04 (3.12)**	**7.70 (3.49)**	**8.30 (3.00)**	***F* = 5.03 (0.007)**
Physical development (*M, SD*)	4.63 (0.49)	4.64 (0.47)	4.64 (0.48)	4.64 (0.48)	*F* = 0.02 (0.098)

*Note*: Bold text = significant at α = 0.05. Participant characteristics at the 17 year follow‐up. All analyses except χ^*2*^ tests were weighted by probability of trajectory membership. Brackets in the final column contain *p* values. Proportion of participants in each income bracket reported in Table S2.

Abbreviations: *M* = mean, *SD* = standard deviation.

**TABLE 4:** Participant characteristics, self‐reported sleep problems and epigenetic age acceleration at the 17 year follow‐up among participants with a total self‐reported sleep problem score (*n* = 891).

Participant characteristic	Self‐reported sleep problems	GrimAA	ExtrinsicAA	HorvathAA	IntrinsicAA	PhenoAA	SkinAA
Sex (female; *b*, *p*)	**0.85 (<0.0001)**	**−0.62 (0.001)**	**−1.43 (<0.0001)**	**−0.81 (0.001)**	**−0.52 (0.03)**	**1.56 (<0.0001)**	**0.71 (<0.0001)**
Age (*r*, *p*)	0.09 (0.008)	**−0.10 (0.004)**	**−0.13 (0.0001)**	**−0.07 (0.049)**	−0.04 (0.27)	**−0.11 (0.001)**	−0.05 (0.15)
BMI (*r*, *p*)	**0.12 (0.0005)**	**0.13 (0.0002)**	**0.11 (0.0007)**	0.06 (0.08)	0.05 (0.11)	**0.19 (<0.0001)**	0.04 (0.24)
Depressive symptoms (*r*, *p*)	**0.61 (<0.0001)**	0.02 (0.50)	−0.02 (0.61)	0.06 (0.09)	**0.07 (0.049)**	**0.10 (0.004)**	0.05 (0.16)
Ethnicity (*F*, *p*)	0.61 (0.66)	1.51 (0.20)	**4.75 (0.0008)**	1.79 (0.13)	1.02 (0.39)	**4.33 (0.002)**	1.16 (0.33)
Family income (*r*, *p*)	**−0.09 (0.01)**	**−0.16 (<0.0001)**	**−0.09 (0.01)**	0.01 (0.71)	0.02 (0.51)	**−0.11 (0.003)**	−0.04 (0.29)
Physical development (*r*, *p*)	−0.06 (0.08)	**0.07 (0.04)**	0.04 (0.20)	0.03 (0.32)	0.04 (0.27)	−0.04 (0.19)	−0.06 (0.08)
Smoking (past 4 weeks; *b*, *p*)	**0.93 (<0.0001)**	**0.99 (<0.0001)**	**1.36 (0.002)**	0.26 (0.38)	0.17 (0.56)	**1.47 (0.001)**	0.04 (0.82)

*Note*: All variables obtained at the 17 year follow‐up. Bold text = significant at α = 0.05.

### 3.2 Agreement between the Child Behaviour Checklist and the Youth Self Report at age 17

Cohen's κ ranged from 0.15 to 0.21 for nominally equivalent self‐ and parent‐reported sleep items at age 17, representing poor to fair agreement using the benchmarks recommended by Landis and Koch^18^ (Table S3). Additionally, the mean total self‐reported sleep problem score at age 17 (*M* = 2.51) was more than three times the size of the nominally equivalent parent‐reported score at the same age (*M* = 0.76, paired *t* = 25.20, *p* <0.0001).

### 3.3 Sleep trajectories from age 5 to 17 and epigenetic age acceleration at age 17

GrimAA was the only measure of EAA to trend in the hypothesised direction across trajectories, with a higher mean in the moderate trajectory than in the minimal trajectory, and a higher mean in the persistent trajectory than in the moderate trajectory (Figure S1 and Table S4). As shown in Table 5, there were no statistically significant differences in EAA between the trajectories. Unweighted sensitivity analyses excluding participants with a <80% probability of membership to their assigned trajectory did not yield substantially different results (Table S6).

**TABLE 5:** Analyses of variance predicting epigenetic age acceleration at age 17 from sleep trajectory from age 5 to 17.

Model	GrimAA	ExtrinsicAA	HorvathAA	IntrinsicAA	PhenoAA	SkinAA
Age‐adjusted	0.05 (0.95)	0.78 (0.46)	0.32 (0.72)	0.34 (0.71)	0.72 (0.49)	0.15 (0.86)
Demographic	0.73 (0.48)	0.18 (0.84)	1.21 (0.30)	0.85 (0.43)	0.70 (0.50)	0.59 (0.55)
Lifestyle	1.80 (0.17)	0.31 (0.73)	0.88 (0.42)	0.32 (0.72)	1.47 (0.23)	0.25 (0.78)
Fully‐adjusted	1.50 (0.22)	0.87 (0.42)	0.93 (0.40)	0.47 (0.62)	1.35 (0.26)	0.20 (0.82)

*Note*: *F* with *p* in brackets. All models were weighted by probability of trajectory membership.

### 3.4 Self‐reported sleep problems and epigenetic age acceleration at age 17

As shown in Table 6, no measures of EAA were significantly related to self‐reported sleep problem score at the 17 year follow‐up in the age‐adjusted model. The relationship between sleep problem score and IntrinsicAA became significant after adjusting for demographic factors, representing an increase of 14% of a year (51 days) per point on the YSR (scored from 0 to 10; Table 6). The effect was largely unchanged after adding lifestyle factors to the model, but it became weaker and non‐significant after further adjusting for depressive symptoms and physical development. There were no other associations between self‐reported sleep problem score and EAA.

**TABLE 6:** Cross‐sectional linear regression predicting epigenetic age acceleration from self‐reported sleep problem score at age 17.

EAA	Age‐adjusted				Demographic				Lifestyle				Fully‐adjusted			
	*b*	95% CI		*p*	*b*	95% CI		*p*	*b*	95% CI		*p*	*b*	95% CI		*p*
GrimAA	0.0008	−0.10	0.10	0.99	0.04	−0.06	0.15	0.41	−0.01	−0.11	0.10	0.86	−0.05	−0.18	0.08	0.43
ExtrinsicAA	−0.16	−0.34	0.02	0.07	−0.05	−0.25	0.14	0.58	−0.16	−0.36	0.04	0.11	−0.20	−0.04	0.05	0.11
HorvathAA	0.06	−0.06	0.19	0.30	0.13	−0.003	0.27	0.06	0.12	−0.02	0.26	0.08	0.06	−0.12	0.23	0.52
IntrinsicAA	0.10	−0.02	0.21	0.10	**0.14**	**0.01**	**0.27**	**0.033**	**0.14**	**0.007**	**0.27**	**0.04**	0.08	−0.09	0.24	0.35
PhenoAA	0.14	−0.04	0.33	0.13	0.08	−0.13	0.28	0.46	−0.01	−0.22	0.19	0.91	−0.09	−0.35	0.17	0.51
SkinAA	0.02	−0.06	0.09	0.62	−0.004	−0.08	0.08	0.91	−0.01	−0.09	0.07	0.82	−0.06	−0.16	0.05	0.28

*Note*: Bold text = significant at α = 0.05. See Table 2 for the covariates in each model.

Abbreviations: *b* = unstandardised coefficient for sleep problems, CI = confidence interval. [Correction added on 14 March 2023, after first online publication: The colunm headings have been corrected in this version.]

### 3.5 Individual sleep problems and intrinsic epigenetic age acceleration at age 17

Separate analyses were performed predicting IntrinsicAA from each item on the YSR at age 17, to investigate the extent to which each item contributed to the relationship with the total self‐reported score in the lifestyle model. As reported in Table S5, “I feel overtired” was the only item that showed a significant association with IntrinsicAA, predicting an increase of 40% of a year (146 days) per point on the YSR (scored from 0 to 2).

### 3.6 Sensitivity analyses

#### 3.6.1 Overtiredness and intrinsic epigenetic age acceleration at age 17

To investigate the extent to which the cross‐sectional relationship between total self‐reported sleep problem score and IntrinsicAA at age 17 in the lifestyle model depended on the overtired item, we performed a sensitivity analysis predicting IntrinsicAA from the total score calculated without that item. The modified total score was not significantly associated with IntrinsicAA, although the regression coefficient was unaffected (unmodified score: *b* = 0.14, *p* = 0.04; modified score: *b* = 0.14, *p* = 0.12).

#### 3.6.2 Adjusting for physical development and depressive symptoms separately at age 17

We added physical development and depressive symptoms at age 17 to the lifestyle model separately to investigate their effect on the significant associations between overtiredness and IntrinsicAA and total self‐reported sleep problem score and IntrinsicAA. Neither relationship was affected by adding physical development without depressive symptoms (Table S7). Both associations became weaker and non‐significant after adding depressive symptoms without physical development, however (overtiredness: *b* = 0.40 changed to 0.25, *p* = 0.04 to 0.24; total score: *b* = 0.14 to 0.08, *p* = 0.04 to 0.34). Consistent with possible confounding, depressive symptoms were positively related to overtiredness (*r* = 0.46, *p* <0.0001), self‐reported sleep problem score, and IntrinsicAA (Table 4).

## 4 DISCUSSION

We investigated if parent‐reported sleep trajectories from age 5 to 17, and self‐reported sleep problems at age 17, were associated with EAA at age 17 in a representative cohort of young Australians from the Raine Study. There was no evidence for a relationship between the sleep trajectories and EAA. At the 17 year follow‐up, IntrinsicAA was weakly associated with current sleep problem score, predicting an increase of just over 7 weeks per point on the questionnaire (scored from 0 to 10) after adjusting for demographic and lifestyle factors. There was no evidence for a relationship between sleep problem score and any other measure of EAA. IntrinsicAA has been associated with minimally heightened risk of cancer,^19^ metabolic syndrome,^20^ and all‐cause mortality.^6^ Associations with this biomarker may therefore have small but non‐trivial implications for long‐term health.

The relationship between self‐reported sleep problem score and IntrinsicAA became weaker and non‐significant after adjusting for depressive symptoms. Consistent with confounding, depressive symptoms were positively related to both sleep problem score and IntrinsicAA. Overtiredness was the only item composing the total score to show a significant independent association with IntrinsicAA, and the total score was not significantly related to IntrinsicAA when calculated without the overtired item. The relationship between the total score and IntrinsicAA may therefore represent a link between overtiredness and IntrinsicAA. While it is possible that overtiredness was associated with IntrinsicAA because poorer sleepers (e.g., participants with sleep disorders) were more likely to say they were overtired, we were not able to interpret our finding as clear evidence for a relationship between sleep and IntrinsicAA. Any number of factors apart from sleep may have made participants more likely to report overtiredness, including (but not limited to) life stressors (e.g., academic pressure) and depressive symptoms.

We failed to replicate the only previously‐reported finding on sleep and EAA in adolescence. Unlike Carskadon et al.,^12^ we observed no evidence for a relationship between sleep and GrimAA or HorvathAA in late adolescence. Contrasting findings may be due to sample size, as we included substantially more participants than the previous study (*N* > 700 fully adjusted, compared to *N* = 12). Different covariates and measures of sleep may also have contributed, as Carskadon et al.^12^ did not control for depressive symptoms and used a sleep diary measure. Our null result also contrasts with evidence for a relationship between sleep and EAA in middle‐aged and older adults.^8^, ^9^, ^11^ It may be that sleep is not reliably linked with EAA in late adolescence, but it becomes more strongly linked with age. Age differences could be associated with changes in epigenetic ageing, sleep behaviour and physiology, and age‐related environmental factors, such as expectations regarding school and work attendance.^5^, ^21^ Further research in younger people will be needed to determine if mixed findings may reflect a true age difference in the relationship between sleep and EAA.

It is of note that we observed effects for IntrinsicAA, but not HorvathAA. IntrinsicAA adjusts for the estimated abundance of various white blood cell types and represents epigenetic differences that vary with calendar age across a wide range of cell and tissue types.^6^ HorvathAA is the same measure without adjusting for cell abundance, suggesting cell composition may have suppressed the effects observed for IntrinsicAA. Because the observed effects were only visible after adjusting for white blood cell composition, it is not likely that they were attributable to it.

There is little research on the biological meaning of IntrinsicAA, but evidence regarding its unadjusted equivalent (HorvathAA) may help to interpret our findings. A recent in vitro study suggests several of the mechanisms proposed in a recent review of sleep and biological age^4^ may not be clearly represented by HorvathAA, including cellular senescence, DNA damage, telomere attrition, and altered mitochondrial function.^22^ It is also unlikely that IntrinsicAA represents inflammation, as it is not associated with inflammatory markers in Gen2 of the Raine Study.^23^ Our pattern of results would not be compatible, therefore, with the specific pathways proposed in the review. The general premise of altered or impaired cellular function may nevertheless hold, as HorvathAA may capture age‐related differences in haematopoietic stem cells and methylomic stability (the integrity of cell‐specific patterns of DNA methylation) that would not be controlled for by adjustments made for white blood cell composition.^24^, ^25^ Recently, an experimental study by McAlpine et al.^26^ in humans and mice revealed evidence to suggest sleep fragmentation may accelerate the gradual decay of the haematopoietic system, leading to impaired immune function. If the overtired measure in the present study captures some aspect of poor sleep health, the relationship with IntrinsicAA may represent the impact of sleep on the haematopoietic system, potentially through changes to the methylome. Given the ambiguity of the overtired measure, however, and the lack of evidence for a relationship with more explicit sleep parameters such as trouble sleeping, we were not able to draw this conclusion. Putting aside the potential role of sleep, the observed relationships may represent a link between overtiredness, depressive symptoms, and the state of the haematopoietic system in late adolescence. These findings reflect a growing body of literature suggesting mental health in young people may be associated with age‐related changes to the blood methylome, with potential implications for long‐term health.^27^

Our findings must be interpreted in light of various limitations. As with all observational research, there are potentially important confounders that were not accounted for. Antidepressants, for example, may have impacted our findings, having been associated with HorvathAA in a previous study.^28^ Our study may also be limited by the ethnic composition of the sample, which is primarily white Australian – it is not known if the results are generalisable to other ethnicities. It is important to note, too, that our significant findings would not have withstood correction for multiple testing. Unfortunately, standard adjustments such as the Bonferroni correction would have resulted in an unfeasible loss of statistical power.

Our study is also limited by the use of parent‐ and self‐report measures of sleep, which may be less suited to investigate the biology of sleep than objective measures such as polysomnography. While aspects of the sleep metrics from the parent‐report CBCL correlate with sleep disorder diagnosis in similarly aged participants,^15^ researchers investigating the CBCL in relation to objective measures of sleep more broadly have reported little evidence for a relationship.^29^ Our findings also indicate possible parent underreporting, with a total score on the CBCL less than one‐third of the nominally equivalent score on the YSR. Additionally, we observed poor to fair agreement between each sleep item on the questionnaire and its self‐report equivalent at the 17 year follow‐up. Low agreement may represent poor validity for the CBCL at age 17, assuming adolescents are in a better position to report on their sleep than their parents. It is important to note, however, that by late adolescence, parents may have less awareness of sleep behaviours relative to earlier childhood. Future studies may use objective measures of sleep and child‐report questionnaires, which are validated in children as young as eight.^30^

This study assessed self‐ and parent‐reported sleep and EAA in late adolescence, including long‐ and short‐term measures of sleep health, six measures of EAA, and a wide range of behavioural and demographic covariates. There was no evidence for a relationship between long‐term patterns of parent‐reported sleep or self‐reported sleep and EAA in late adolescence. There was, however, support for a relationship between overtiredness, depressive symptoms, and higher IntrinsicAA in late adolescence. Our findings highlight the need to consider mental health as a potential confounding variable in future research on sleep and EAA, particularly if subjective measures of sleep are used. Additionally, further research on younger participants will be needed to determine if mixed findings in adults and adolescents may reflect a true age difference in the relationship between sleep and EAA.

## AUTHOR CONTRIBUTIONS

David Balfour, Sarah Cohen‐Woods, and Amy C. Reynolds conceived of the study design. David Balfour, Sarah Cohen‐Woods, Amy C. Reynolds, and Sian Wanstall prepared the study and data request. Joanne A. McVeigh was involved in the development of the trajectory variable. Rae‐Chi Huang performed DNA methylation data processing and quality control procedures. Phillip E. Melton calculated the epigenetic age and cell abundance estimates, which David Balfour used to calculate epigenetic age acceleration. David Balfour performed the analyses and wrote the initial manuscript. All authors edited the manuscript and offered feedback, which David Balfour responded to. All authors read and approved the final manuscript.

## CONFLICT OF INTEREST STATEMENT

None.

## CONSENT TO PARTICIPATE

Caregivers provided written consent for their Gen2 children to participate in the study until they were old enough to provide their own consent.

## Supporting information


Figure S1

Figure S2

Data S1

## Data Availability

Data access is subject to restrictions imposed in order to protect participant privacy. All researchers using Raine Study data must sign a data access agreement stipulating that data may not be released to anyone other than the investigators of the approved project. Additional details regarding data access are available from https://rainestudy.org.au/.
