# Supplementary figures and images for: Childhood sleep health and epigenetic age acceleration in late adolescence: Cross‐sectional and longitudinal analyses
Source: Acta Paediatr. 2023 Mar 3;112(5):1001–10. doi: 10.1111/apa.16719 (PMC10952569; doi:10.1111/apa.16719)

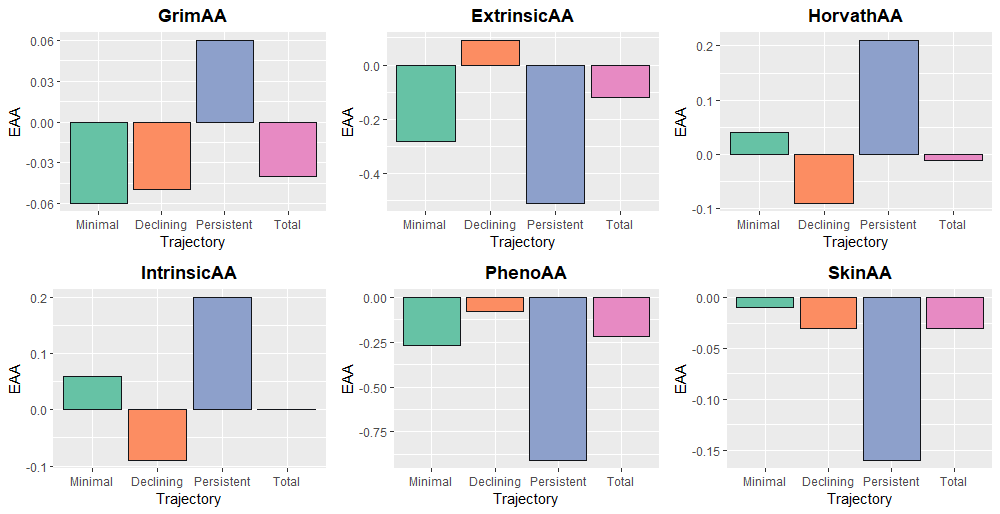

Supplement: Supplementary file 1 — Figure S1 [file APA-112-1001-s001.png]

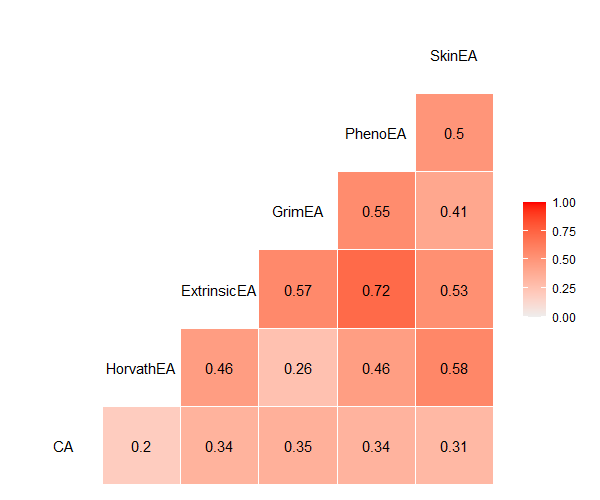

Supplement: Supplementary file 2 — Figure S2 [file APA-112-1001-s002.png]
