# Supplementary material for: Childhood sleep health and epigenetic age acceleration in late adolescence: Cross‐sectional and longitudinal analyses
Source: Acta Paediatr. 2023 Mar 3;112(5):1001–10. doi: 10.1111/apa.16719 (PMC10952569; doi:10.1111/apa.16719)
Supplement: Supplementary file 3 — Data S1 [file APA-112-1001-s003.docx]

**Childhood sleep health and epigenetic age acceleration in adolescence: Cross-sectional and longitudinal analyses**

David Balfour^1^, Phillip E. Melton^2,3^, Joanne A. McVeigh^4,5^, Rae-Chi Huang^6^, Peter R. Eastwood^7^, Sian Wanstall^7^, Amy C. Reynolds^7^, and Sarah Cohen-Woods^1,8^

^1^Discipline of Psychology, Flinders University, South Australia

^2^Menzies Institute for Medical Research, College of Health and Medicine, University of Tasmania, Tasmania

^3^School of Global and Population Health, the University of Western Australia, Nedlands

^4^Curtin School of Allied Health, Curtin University, Western Australia

^5^Movement Physiology Laboratory, School of Physiology, University of Witwatersrand, South Africa

^6^Nutrition and Health Innovation Research Institute (NHIRI), Edith Cowan University, Western Australia

^7^Flinders Health and Medical Research Institute (Sleep Health), Flinders University, South Australia

^8^Flinders Centre for Innovation in Cancer, Flinders University, South Australia

**APPENDIX**

[Contents 2](#_Toc124196716)

[Epigenetic age acceleration 4](#_Toc124196717)

[Definition of epigenetic age acceleration 4](#_Toc124196718)

[Relationship between epigenetic age and calendar age 5](#_Toc124196719)

[Covariates 6](#_Toc124196720)

[Agreement between the Child Behavior Checklist and the Youth Self Report 7](#_Toc124196721)

[Missing data 8](#_Toc124196722)

[Number of participants included in each model 8](#_Toc124196723)

[Additional analyses 9](#_Toc124196724)

[Alternative method for handling missing data for depressive symptoms 9](#_Toc124196725)

[Overtiredness and white blood cell composition 9](#_Toc124196726)

[White blood cell count as a possible mediator 10](#_Toc124196727)

[Supplementary tables 11](#_Toc124196728)

[Supplementary figures 22](#_Toc124196729)

# Epigenetic age acceleration

## Definition of epigenetic age acceleration

The following measures of EAA were defined as the residual from regressing epigenetic age on calendar age at the 17 year follow-up: HorvathAA,^[[1]](#footnote-1)^ SkinAA,^[[2]](#footnote-2)^ ExtrinsicAA,^[[3]](#footnote-3),^^[[4]](#footnote-4)^ GrimAA,^[[5]](#footnote-5)^ and PhenoAA.^[[6]](#footnote-6)^ Following the approach used by Chen et al.,^4^ we defined IntrinsicAA as the residual from regressing epigenetic age on calendar age plus DNA methylation-based estimates of the abundance of the following white blood cell types: CD4+ T cells, natural killer cells, monocytes, granulocytes, plasmablasts, naive CD8+ T cells, and CD8+CD28-CD45RA- T cells. The last three abundance estimates were calculated using the approach developed by Horvath,^1^ and the other estimates were obtained using the approach published by Houseman et al.^^[[7]](#footnote-7)^^ ExtrinsicAA is computed using a measure of epigenetic age that is weighted to include information about DNA methylation-based estimates of the following blood cell types, known to change with age: plasma B cells, late differentiated (CD28–CD45RA–) cytotoxic T cells, and naive (CD45RA+CCR7+) cytotoxic T cells.^[[8]](#footnote-8)^

## Relationship between epigenetic age and calendar age

Median absolute error with calendar age is often used to represent the precision of epigenetic age,1 as epigenetic clocks are usually designed to estimate calendar age or an age-related phenotype or outcome. Estimates from clocks that do not only predict calendar age are typically expressed in years, transformed to align with calendar age based on the original data used to construct the clock. Of the three measures developed to estimate calendar age in this study (Table 1), Horvath’s skin and blood clock (SkinEA) was the most precisely calibrated, with a median absolute error of just 1.88 years (Table S1). This finding aligns with previous research demonstrating the clock’s superior ability to predict calendar age in children and adolescents.^[[9]](#footnote-9),^^[[10]](#footnote-10)^ A close relationship with calendar age is not always desirable, however, as there is an inverse relationship between the ability to predict calendar age and the ability to predict mortality.^[[11]](#footnote-11)^ This relationship occurs because epigenetic age only provides unique information about health to the extent that it deviates from calendar age; clocks that are more closely aligned with calendar age provide less information above and beyond calendar age by definition.^[[12]](#footnote-12)^ Clocks designed to estimate mortality and age-related phenotypes are usually less strongly related to calendar age but more strongly related to health.^[[13]](#footnote-13)^ Consistent with this pattern, DNA methylation GrimAge (GrimEA) and DNA methylation PhenoAge (PhenoEA) had the highest median absolute error for calendar age (8.59 and 8.15 years, respectively), but they were also the most strongly related to BMI (Table 4). As an additional measure of precision, we calculated bivariate correlations between epigenetic age and calendar age (Figure S2). Correlations ranged from .20 for Horvath’s DNA methylation age (Horvath EA) to .35 for Grim EA. These somewhat low values are likely due to the low spread of calendar age in the sample, making median absolute error a more suitable measure.1

# Covariates

Data were gathered on the following covariates at age 17: body mass index (BMI),^^[[14]](#footnote-14)^,^[[15]](#footnote-15)^^ depressive symptoms, ethnicity,^^[[16]](#footnote-16)^,^[[17]](#footnote-17)^^ family income,^^[[18]](#footnote-18)^,^[[19]](#footnote-19)^^ physical development, sex,^^[[20]](#footnote-20)^,^[[21]](#footnote-21)^^ and smoking.^^[[22]](#footnote-22)^,^5 Data on ethnicity and sex were obtained through self-report. Height and weight were measured and used to calculate BMI (weight in kg/height in m^2^). Total family income before tax was obtained through parent report. The Tanner scale was used to assess physical development, with adolescents asked to choose images representing their level of breast and genital development. The genital options were modified to exclude the fifth and final stage of development. Tanner stage for female participants was included as the mean score for breast and genital development. Depressive symptoms were assessed using the Beck Depression Inventory for Youth.^[[23]](#footnote-23)^ Smoking was assessed through self-report, with Gen2 participants asked if they had smoked cigarettes in the past 4 weeks.

# Agreement between the Child Behavior Checklist and the Youth Self Report

We investigated the level of agreement between self- and parent-reported sleep problem scores at the 17 year follow-up to informally assess the level of response bias in the parent-reported scores that were used to construct the trajectories. Following the approach used by Fatima et al.,^[[24]](#footnote-24)^ we constructed a binary variable for each self- and parent-reported sleep item, with “Not true (as far as you know)” coded as 0, and “somewhat or sometimes true” and “very true or often true” both coded as 1. Cohen’s κ and percent agreement were then calculated to represent the level of agreement between the binarised self- and parent-reported items (Table S3). Cohen’s κ ranged from .15 to .21, representing poor to fair agreement using the benchmarks recommended by Landis and Koch.^^[[25]](#footnote-25)^^ Percent agreement ranged from 54.57% to 73.45%, indicating participants and their parent agreed on the presence or absence of a sleep problem approximately one half to three quarters of the time.

For comparison with the total sleep problem score on the Youth Self Report (YSR), we calculated a 5-item Child Behavior Checklist (CBCL) score at age 17, including all items except “talks or walks in sleep”. We used a paired *t*-test to compare the mean self-reported score with the mean five-item parent-reported score among participants who had a score for both questionnaires. The mean score on the YSR (*M* = 2.51, *SD* = 2.00) was more than three times the size of the mean (nominally equivalent, five-item) score on the CBCL (*M* = 0.76, *SD* = 1.30, *t* = 25.20, *p* < .0001).

# Missing data

## Number of participants included in each model

After listwise deletion, 1,053 participants from the total sample of 1,192 were included in age-adjusted analyses of variance predicting EAA from sleep trajectory, due to missing trajectory data (139 participants excluded). A total of 805 participants were included in the demographic-adjusted analysis of variance, due to greater missing data for the additional covariates (387 participants excluded). 711 participants were included in the equivalent lifestyle model (481 participants excluded), and 674 participants were included in the equivalent fully-adjusted model (518 participants excluded).

Total self-reported sleep problem scores were calculated for participants with no more than two missing items. 23 participants had one missing item and 8 participants had two missing items. Missing items were coded as 0 (“not true”). After excluding participants with more than two missing items, 891 participants were included in age-adjusted linear regression analyses predicting EAA from self-reported sleep problem score (301 participants excluded). A total of 769 participants were included in each demographic model using self-reported sleep problem score, due to greater missing covariate data (423 participants excluded). 758 participants were included in the equivalent lifestyle models (434 participants excluded), and 716 participants were included in the equivalent fully-adjusted models (476 participants excluded). See Table S8 for the number of participants missing each variable.

**Demographic comparison between participants with and without sleep data**

We compared demographic variables by missing data status for sleep trajectory and self-reported sleep problem score, to assess the possibility that missing data would impact the generalisability of our findings (Table S9). Sex and ethnicity did not differ between participants with and without missing sleep data (*p* > .05). Family income was lower both among participants who were not assigned to a sleep trajectory (χ^2^ = 23.22, *p* = .02) and among participants who did not have a total self-reported sleep problem score (χ^2^ = 22.51, *p* =.02). The difference in family income was not large, being roughly equivalent to 1 of 12 income brackets (Table S9). The observed relationship between family income and missing data resembles the pattern of attrition bias seen in the overall cohort, where there were slightly more dropouts among participants who were more socioeconomically disadvantaged.^[[26]](#footnote-26)^ Adolescent participants at the 17 year follow-up were otherwise representative of the general population in Western Australia.26 Because the biases relating to socioeconomic status were small, it is not likely they would have a substantial impact on the generalisability of our findings.

# Additional analyses

## Alternative method for handling missing data for depressive symptoms

We performed additional sensitivity analyses to investigate if the cross-sectional relationships between total self-reported sleep problem score, overtiredness, and IntrinsicAA at age 17 may have been attenuated after adding depressive symptoms to the model due to the impact of missing data on statistical power. 758 participants were included in lifestyle-adjusted primary analyses for total score (Table 5) and overtiredness (Table S5). 716 participants were included in primary analyses adding depressive symptoms, due to missing data for the Beck Depression Inventory for Youth. We performed the same analyses with a depressive symptom score calculated for all participants with no more than two missing items, rather than only participants with a response to all 20 items on the questionnaire. As reported in Table S7, this alternative method of dealing with missing data did not affect the association with overtiredness or the association with total self-reported sleep problem score (*n* = 748; overtiredness: *b* = 0.26, *p* = .22; total: *b* = 0.09, *p* = .29).

## Overtiredness and white blood cell composition

Cell composition is a known confounder in DNA methylation analyses, as different cell types have different patterns of DNA methylation.^[[27]](#footnote-27)^ To investigate the potential impact of white blood cell composition on the cross-sectional relationship between overtiredness and IntrinsicAA, we constructed a lifestyle-adjusted regression model predicting HorvathAA from the overtired item at age 17. This enabled us to assess the effect of cell composition because HorvathAA is equivalent to IntrinsicAA without adjustment for white blood cell types. As shown in Table S10, the overtired item was not significantly associated with HorvathAA (*b* = 0.35, *p* = .08).

## White blood cell count as a possible mediator

Previous studies have reported evidence for a relationship between sleep and blood cell counts,^[[28]](#footnote-28)^ and fatigue and blood cell counts.^[[29]](#footnote-29)^ Depressive symptoms have also been associated with blood cell count in several studies.^[[30]](#footnote-30),^^[[31]](#footnote-31),^^[[32]](#footnote-32),^^[[33]](#footnote-33)^ It is therefore plausible that depressive symptoms and/or overtiredness affect EAA through their effect on blood cell counts. We performed a series of exploratory analyses to investigate this possibility. In these analyses, we tested each cell abundance estimate as a mediator between depressive symptoms and HorvathAA, and between overtiredness and HorvathAA. There were no statistically significant indirect effects of depressive symptoms or overtiredness on HorvathAA through the estimates (Table S11).

# Supplementary tables

**Table S1** Median absolute error for epigenetic age and calendar age in the total sample (*N* = 1,192) at the 17 year follow-up

|  | Age | HorvathEA | ExtrinsicEA | GrimEA | PhenoEA | SkinEA |
| --- | --- | --- | --- | --- | --- | --- |
| Age | — | — | — | — | — | — |
| HorvathEA | 5.96 | — | — | — | — | — |
| ExtrinsicEA | 4.52 | 9.23 | — | — | — | — |
| GrimEA | 8.59 | 3.47 | 11.9 | — | — | — |
| PhenoEA | 8.15 | 13.82 | 4.94 | 16.76 | — | — |
| SkinEA | 1.88 | 4.05 | 5.16 | 6.96 | 9.85 | — |

*Note:* EA = epigenetic age.

**Table S2** Family income brackets at age 17 by sleep trajectory and among all participants assigned to a trajectory (*n* = 1,053)

|  | Consistently minimal sleep problems | Moderate but declining sleep problems | Persistent sleep problems | Total | Comparison between trajectories |
| --- | --- | --- | --- | --- | --- |
| Participants (*n*, %) | 470 (44.63) | 504 (47.86) | 79 (7.50) | 1,053 (100) |  |
| Family income (*n*, %) |  |  |  |  | χ^2^ = 26.53 (.23) |
| $1–$8,000 | 4 (0.78) | 1 (0.21) | 3 (0.36) | 4 (0.49) |  |
| $8,001–$16,000 | 16 (3.10) | 10 (2.07) | 16 (1.94) | 15 (1.83) |  |
| $16,001–$25,000 | 33 (6.40) | 23 (4.75) | 41 (4.98) | 41 (5.01) |  |
| $25,001–$30,000 | 23 (4.46) | 17 (3.51) | 35 (4.25) | 36 (4.40) |  |
| $30,001–$35,000 | 16 (3.10) | 13 (2.69) | 25 (3.03) | 27 (3.30) |  |
| $35,001–$40,000 | 14 (2.71) | 15 (3.10) | 24 (2.91) | 25 (3.05) |  |
| $40,001–$50,000 | 47 (9.11) | 42 (8.68) | 75 (9.10) | 67 (8.18) |  |
| $50,001–$60,000 | 46 (8.91) | 37 (7.64) | 65 (7.89) | 59 (7.20) |  |
| $60,001–$70,000 | 39 (7.56) | 29 (5.99) | 56 (6.80) | 56 (6.84) |  |
| $70,001–$78,000 | 32 (6.20) | 41 (8.47) | 62 (7.52) | 63 (7.69) |  |
| $78,001–$104,000 | 99 (19.19) | 91 (18.80) | 153 (18.57) | 158 (19.29) |  |
| $104,001+ | 147 (28.49) | 165 (34.09) | 269 (32.65) | 268 (32.72) |  |

*Note:* Analysis was not weighted by probability of trajectory membership.

**Table S3** Level of agreement between binarised self- and parent-reported sleep problems at age 17

| Problem | Cohen's κ | Percent agreement |
| --- | --- | --- |
| “I have trouble sleeping” | 0.21 | 61.71% |
| “I sleep less than most kids” | 0.11 | 60.59% |
| “I sleep more than most kids during the day and/or night” | 0.17 | 73.45% |
| “I feel overtired” | 0.14 | 54.57% |
| “I have nightmares” | 0.15 | 71.69% |

**Table S4** Epigenetic age acceleration at age 17 by sleep trajectory and among all participants assigned to a trajectory

| EAA | Consistently minimal sleep problems (*n* = 470) | | Moderate but declining sleep problems (*n* = 504) | | Persistent sleep problems (*n* = 79) | | Total (*n* = 1,053) | |
| --- | --- | --- | --- | --- | --- | --- | --- | --- |
|  | *M* | *SD* | *M* | *SD* | *M* | *SD* | *M* | *SD* |
| GrimAA | -0.06 | 2.91 | -0.05 | 3.05 | 0.06 | 2.6 | -0.04 | 2.96 |
| ExtrinsicAA | -0.28 | 5.4 | 0.09 | 5.27 | -0.51 | 5.22 | -0.12 | 5.33 |
| HorvathAA | 0.04 | 3.45 | -0.09 | 3.76 | 0.21 | 3.45 | -0.01 | 3.6 |
| IntrinsicAA | 0.06 | 3.33 | -0.09 | 3.62 | 0.20 | 3.36 | 0.00 | 3.47 |
| PhenoAA | -0.27 | 5.60 | -0.08 | 5.74 | -0.91 | 5.00 | -0.22 | 5.62 |
| SkinAA | -0.01 | 2.17 | -0.03 | 2.26 | -0.16 | 2.25 | -0.03 | 2.22 |

*Note:* *M* = mean, *SD* = standard deviation. Means and standard deviations were weighted by probability of trajectory membership.

**Table S5** Cross-sectional adjusted linear regression predicting intrinsic epigenetic age acceleration from individual self-reported sleep problems at age 17

| Problem | *b* | 95% CI | | *p* |
| --- | --- | --- | --- | --- |
| “I have trouble sleeping” | 0.34 | -0.06 | 0.73 | 0.09 |
| “I sleep less than most kids” | 0.07 | -0.3 | 0.44 | 0.72 |
| “I sleep more than most kids during the day and/or night” | 0.25 | -0.19 | 0.69 | 0.26 |
| **“I feel overtired”** | **0.40** | **0.02** | **0.77** | **0.04** |
| “I have nightmares” | 0.20 | -0.3 | 0.69 | 0.44 |

*Note:* *b* = unstandardised coefficient for individual sleep problem (scored from 0 to 2), CI = confidence interval, bold text = significant at α = .05. Separate analyses were performed for each item. All analyses were lifestyle models, controlling for BMI, calendar age, ethnicity, family income, sex, and smoking.

**Table S6** Unweighted analyses of variance predicting epigenetic age acceleration at age 17 from sleep trajectory from age 5 to 17, excluding participants with < 80% probability of trajectory membership

| Model | Included N (excluded *n*) | GrimAA | ExtrinsicAA | HorvathAA | IntrinsicAA | PhenoAA | SkinAA |
| --- | --- | --- | --- | --- | --- | --- | --- |
| Age-adjusted | 886 (306) | 0.24 (.63) | 0.12 (.74) | 0.03 (.87) | 0.04 (.84) | 0.11 (.74) | 0.12 (.73) |
| Demographic | 571 (234) | 0.67 (.51) | 0.26 (.77) | 1.17 (.31) | 0.90 (.41) | 0.37 (.69) | 1.29 (.28) |
| Lifestyle | 508 (203) | 1.78 (.17) | 0.90 (.41) | 1.01 (.36) | 0.56 (.57) | 1.27 (.28) | 0.97 (.38) |
| Fully-adjusted | 481 (193) | 1.62 (.20) | 1.04 (.35) | 1.11 (.33) | 0.79 (.46) | 1.06 (.35) | 1.08 (.34) |

*Note:* *F* with *p* in brackets. Models were not weighted by probability of trajectory membership.

**Table S7** Cross-sectional adjusted linear regression predicting intrinsic epigenetic age acceleration from total self-reported sleep problem score, including additional covariates at age 17

| Missing data method^a^ | Additional covariate | Predictor | *b* | 95% CI | | *p* | Included (*n*)^c^ |
| --- | --- | --- | --- | --- | --- | --- | --- |
| **Listwise deletion** | **Tanner stage** | **Total sleep problem score** | **0.14** | **0.007** | **0.27** | **.04** | **758** |
| **Listwise deletion** | **Tanner stage** | **Overtiredness** | **0.40** | **0.02** | **0.77** | **.04** | **758** |
| Listwise deletion | Depressive symptoms | Total sleep problem score | 0.08 | -0.08 | 0.25 | .34 | 716 |
| Listwise deletion | Depressive symptoms | Overtiredness | 0.25 | -0.17 | 0.67 | .24 | 716 |
| Exclude if missing > 2 items^b^ | Depressive symptoms | Total sleep problem score | 0.09 | -0.07 | 0.25 | .29 | 748 |
| Exclude if missing > 2 items^b^ | Depressive symptoms | Overtiredness | 0.26 | -0.15 | 0.67 | .22 | 748 |

*Note:* *b* = unstandardised coefficient for predictor, CI = confidence interval, bold text = significant at α = .05. Models controlled for BMI, CA, ethnicity, family income, sex, and smoking, plus the specified additional covariate.

^a^Method for handling missing data for additional covariate.

^b^Exclude participants with more than two missing items on the Beck Depression Inventory for Youth.

^c^Total number of participants included in analysis.

**Table S8** Proportion of participants missing each variable

| Variable | Number and percentage missing among all participants (*n* = 1,192) |
| --- | --- |
| Sex | 0 (0) |
| Age | 0 (0) |
| GrimEA | 0 (0) |
| ExtrinsicEA | 0 (0) |
| HorvathEA | 0 (0) |
| PhenoEA | 0 (0) |
| SkinEA | 0 (0) |
| Ethnicity | 21 (1.76) |
| Family income | 311 (26.09) |
| BMI | 297 (24.92) |
| Smoking | 308 (25.84) |
| Depressive symptoms | 350 (29.36) |
| Physical development | 299 (25.08) |
| Self-reported sleep problems | 301 (25.25) |
| Parent-reported sleep problems | 306 (25.67) |
| Sleep trajectory | 139 (11.66) |

**Table S9** Demographic comparison between participants with and without sleep data

| Characteristic | Sleep trajectory | | | Self-reported sleep problem score | | |
| --- | --- | --- | --- | --- | --- | --- |
|  | With trajectory data (*n* = 1,053) | Without trajectory data (*n* = 139) | Comparison (test statistic, *p*) | With total score (*n* = 891) | Without total score (*n* = 301) | Comparison (test statistic, *p*) |
| Sex (female; *n*, %) | 519 (49.29) | 66 (47.48) | χ^2^ = 0.16 (.69) | 442 (49.61) | 143 (47.51) | χ^2^ = 0.40 (.53) |
| Ethnicity (*n*, %) |  |  | χ^2^ = 6.82 (.15) |  |  | χ^2^ = 6.93 (.14) |
| African or African American | 48 (4.64) | 6 (4.41) |  | 43 (4.91) | 11 (3.73) |  |
| East Asian | 142 (13.72) | 19 (13.97) |  | 131 (14.95) | 30 (10.17) |  |
| European | 798 (77.10) | 101 (74.26) |  | 659 (75.23) | 240 (81.36) |  |
| South Asian | 28 (2.71) | 9 (6.62) |  | 26 (2.97) | 11 (3.73) |  |
| Other | 19 (1.84) | 1 (0.74) |  | 17 (1.94) | 3 (1.02) |  |
| Family income (0 to 11; *M*, *SD*) | 8.30 (3.00) | 7.52 (3.19) | ***F* = 3.87 (.05)^a^; χ^2^ = 23.22 (.02)** | 8.37 (2.93) | 7.26 (3.55) | ***F* = 11.88 (.0006)^a^; χ^2^ = 22.51 (.02)** |

*Note:* *M* = mean, *SD* = standard deviation, bold text = significant at α = .05. Analyses were not weighted by probability of trajectory membership, unless otherwise stated.

^a^Weighted by probability of trajectory membership.

**Table S10** Cross-sectional adjusted linear regression predicting AgeAccelHorvath from individual self-reported sleep problems at age 17

| Problem | *b* | 95% CI | | *p* |
| --- | --- | --- | --- | --- |
| “I have trouble sleeping” | 0.31 | -0.10 | 0.72 | .14 |
| “I sleep less than most kids” | 0.009 | -0.38 | 0.39 | .97 |
| “I sleep more than most kids during the day and/or night” | 0.24 | -0.22 | 0.70 | .30 |
| “I feel overtired” | 0.35 | -0.04 | 0.74 | .08 |
| “I have nightmares” | 0.20 | -0.31 | 0.72 | .44 |

*Note:* AgeAccelHorvath (HorvathAA) has not been adjusted for white blood cell abundance estimates. *b* = unstandardised coefficient for individual sleep problem (scored from 0 to 2), CI = confidence interval, bold text = significant at α = .05. Separate analyses were performed for each item. All analyses were lifestyle models, controlling for BMI, calendar age, ethnicity, family income, sex, and smoking.

**Table S11** Indirect effects of depressive symptoms and overtiredness on AgeAccelHorvath through estimated cell abundance

| Cell | Depressive symptoms | | | | Overtiredness | | | | |
| --- | --- | --- | --- | --- | --- | --- | --- | --- | --- |
|  | *b* | 95% CI | | *p* | *b* | 95% CI | | | *p* |
| CD4+ T | 0.005 | -0.002 | 0.013 | .18 | -0.07 | | -0.16 | 0.014 | .10 |
| CD8+CD28-CD45RA- T | 0.0006 | -0.008 | 0.01 | .89 | -0.05 | | -0.15 | 0.06 | .37 |
| Granulocyte | 0.004 | -0.0008 | 0.008 | .11 | -0.03 | | -0.08 | 0.01 | .18 |
| Monocyte | 0.0006 | -0.003 | 0.005 | .78 | 0.003 | | -0.04 | 0.04 | .90 |
| Naive CD8+ T | -0.001 | -0.007 | 0.004 | .61 | -0.05 | | -0.11 | 0.007 | .08 |
| Natural killer | -0.004 | -0.008 | 0.001 | .12 | -0.01 | | -0.07 | 0.05 | .74 |
| Plasmablast | 0.003 | -0.002 | 0.009 | .26 | -0.02 | | -0.07 | 0.04 | .57 |

*Note:* Mediation models with depressive symptom score or overtiredness as the independent variable, estimated cell abundance as the mediator, and AgeAccelHorvath (HorvathAA) as the dependent variable. Separate models were created for each cell type. Normal-based confidence intervals were obtained using bootstrapping with 1000 replications. *b* = unstandardised coefficient for the indirect effect of depressive symptoms or overtiredness on HorvathAA through estimated cell abundance, CI = confidence interval. All analyses were lifestyle models, controlling for BMI, calendar age, ethnicity, family income, sex, and smoking.

# Supplementary figures

**Figure S1** Epigenetic age acceleration at age 17 by sleep trajectory and among all participants assigned to a trajectory (*n* = 1,053)


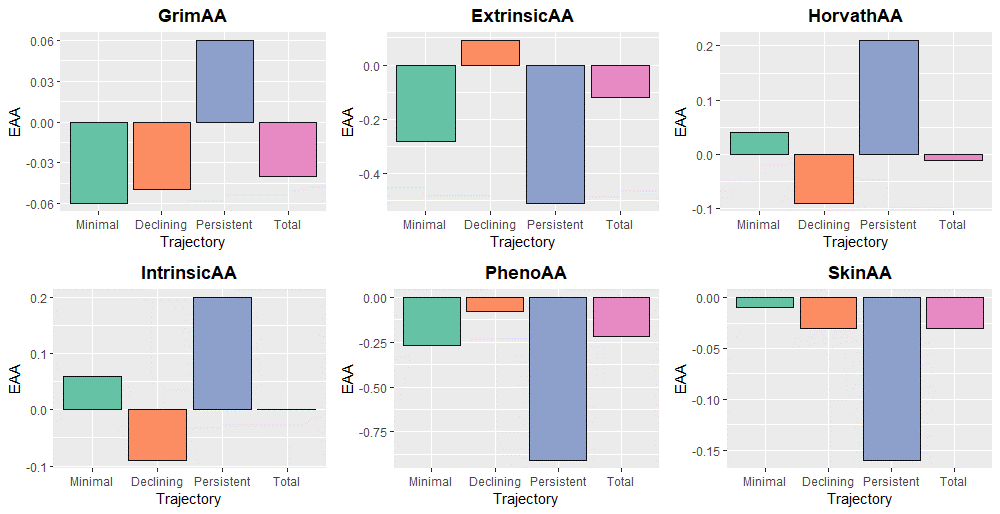


*Note:* Bars represent mean EAA, weighted by probability of trajectory membership. Standard deviations are not included in the figure as they were far larger than the absolute means and would have obscured the direction of differences between the trajectories. Precise means and standard deviations are reported in Table S4.

**Figure S2** Pearson's *r* for epigenetic age and calendar age at the 17 year follow-up


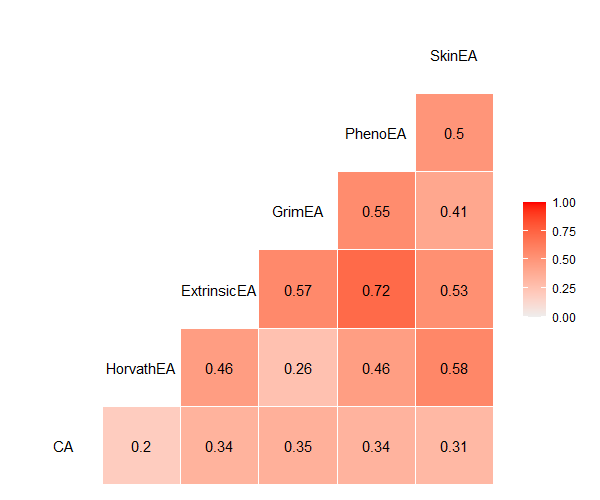


*Note:* CA = calendar age, EA = epigenetic age.

1. . Horvath S. DNA methylation age of human tissues and cell types. *Genome Biol*. 2013;14:3156. <https://doi.org/10.1186/gb-2013-14-10-r115>. [↑](#footnote-ref-1)
2. . Horvath S, Oshima J, Martin GM, et al. Epigenetic clock for skin and blood cells applied to Hutchinson Gilford Progeria Syndrome and ex vivo studies. *Aging (Albany NY)*. 2018;10(7):1758-1775. <https://doi.org/10.18632/aging.101508>. [↑](#footnote-ref-2)
3. . Hannum G, Guinney J, Zhao L, et al. Genome-wide methylation profiles reveal quantitative views of human aging rates. *Mol Cell*. 2013;49(2):359-67. <https://doi.org/10.1016/j.molcel.2012.10.016>. [↑](#footnote-ref-3)
4. . Chen BH, Marioni RE, Colicino E, Peters MJ, Ward-Caviness CK, Tsai PC, et al. DNA methylation-based measures of biological age: meta-analysis predicting time to death. *Aging (Albany NY)*. 2016;8(9):1844-1865. <https://doi.org/10.18632/aging.101020>. [↑](#footnote-ref-4)
5. . Lu AT, Quach A, Wilson JG, et al. DNA methylation GrimAge strongly predicts lifespan and healthspan. *Aging (Albany NY)*. 2019;11(2):303-327. <https://doi.org/10.18632/aging.101684>. [↑](#footnote-ref-5)
6. . Levine ME, Lu AT, Quach A, et al. An epigenetic biomarker of aging for lifespan and healthspan. *Aging (Albany NY)*. 2018;10(4):573-591. <https://doi.org/10.18632/aging.101414>. [↑](#footnote-ref-6)
7. . Houseman EA, Accomando WP, Koestler DC, et al. DNA methylation arrays as surrogate measures of cell mixture distribution. *BMC Bioinform*. 2012;13:86. <https://doi.org/10.1186/1471-2105-13-86>. [↑](#footnote-ref-7)
8. . Carroll JE, Irwin MR, Levine M, et al. Epigenetic aging and immune senescence in women with insomnia symptoms: findings from the Women’s Health Initiative Study. *Biol Psychiatry*. 2017;81(2):136-144. <https://doi.org/10.1016/j.biopsych.2016.07.008>. [↑](#footnote-ref-8)
9. . Kling T, Wenger A, Carén H. Epigenetic clock for skin and blood cells applied to Hutchinson Gilford Progeria Syndrome and ex vivo studies DNA methylation-based age estimation in pediatric healthy tissues and brain tumors. *Aging (Albany NY)*. 2020;12(1):21037-21056. <https://doi.org/10.18632/aging.202145>. [↑](#footnote-ref-9)
10. . Prado-Bert P, Ruiz-Arenas C, Vives-Usano M, et al. The early-life exposome and epigenetic age acceleration in children. *Environ Int*. 2021;155:106683. <https://doi.org/10.1016/j.envint.2021.106683>. [↑](#footnote-ref-10)
11. . Zhang Q, Vallerga CL, Walker RM, et al. Improved precision of epigenetic clock estimates across tissues and its implication for biological ageing. *Genome Med*. 2019;11:54. <https://doi.org/10.1186/s13073-019-0667-1>. [↑](#footnote-ref-11)
12. . Klemera P, Doubal S. Epigenetic biomarkers for biological age. *Mech Ageing Dev*. 2006;127(3):240-248. <https://doi.org/10.1016/j.mad.2005.10.004>. [↑](#footnote-ref-12)
13. . Horvath S, Raj K. DNA methylation-based biomarkers and the epigenetic clock theory of ageing. *Nat Rev Genet*. 2014;19:371-384. <https://doi.org/10.1038/s41576-018-0004-3>. [↑](#footnote-ref-13)
14. . Kresovich JK, Garval EL, Lopez AMM, Xu Z, Niehoff NM, White AJ. Associations of body composition and physical activity level with multiple measures of epigenetic age acceleration. *Am J Epidemiol*. 2020;190(6):984-993. <https://doi.org/10.1093/aje/kwaa251>. [↑](#footnote-ref-14)
15. . Krističević T, Štefan L, Sporiš G. The associations between sleep duration and sleep quality with body-mass index in a large sample of young adults. *Int J Environ Res Public Health*. 2018;15(4):758. <https://doi.org/10.3390/ijerph15040758>. [↑](#footnote-ref-15)
16. . Grandner MA, Williams NJ, Knutson KL, Roberts D, Jean-Louis G. Sleep disparity, race/ethnicity, and socioeconomic position. *Sleep Med*. 2016;18:7-18. <https://doi.org/10.1016/j.sleep.2015.01.020>. [↑](#footnote-ref-16)
17. . Horvath S, Gurven M, Levine ME, et al. An epigenetic clock analysis of race/ethnicity, sex, and coronary heart disease. *Genome Biol*. 2016;17:171. <https://doi.org/10.1186/s13059-016-1030-0>. [↑](#footnote-ref-17)
18. . Felden ÉPG, Leite CR, Rebelatto CF, Andrade RD, Beltrame TS. Sleep in adolescents of different socioeconomic status: a systematic review. *Rev Paul Pediatr*. 2015;33(4):467-473. <https://doi.org/10.1016/j.rppede.2015.08.009>. [↑](#footnote-ref-18)
19. . Fiorito G, Polidoro S, Dugué PA, et al. Social adversity and epigenetic aging: a multi-cohort study on socioeconomic differences in peripheral blood DNA methylation. *Sci Rep*. 2017;7:16266. <https://doi.org/10.1038/s41598-017-16391-5>. [↑](#footnote-ref-19)
20. . Crimmins EM, Thyagarajan B, Levine ME, Weir DR, Faul J. Associations of age, sex, race/ethnicity, and education with 13 epigenetic clocks in a nationally representative U.S. sample: the Health and Retirement Study. *J Gerontol A Biol Sci*. 2021;76(6):1117-1123. <https://doi.org/10.1093/gerona/glab016>. [↑](#footnote-ref-20)
21. . Mong JA, Cusmano DM. Sex differences in sleep: impact of biological sex and sex steroids. *Philos Trans R Soc B: Biol Sci*. 2016;371:20150110. <https://doi.org/10.1098/rstb.2015.0110>. [↑](#footnote-ref-21)
22. . Jaehne A, Unbehaun T, Feige B, Lutz UC, Batra A, Riemann D. How smoking affects sleep: a polysomnographical analysis. *Sleep Med*. 2012;13(10):1286-1292. <https://doi.org/10.1016/j.sleep.2012.06.026>. [↑](#footnote-ref-22)
23. . Beck JS, Beck AT, Jolly JB. *Beck Youth Inventories of Emotional and Social Impairment*. Psychological Corporation; 2001. [↑](#footnote-ref-23)
24. . Fatima Y, Doi SAR, O’Callaghan M, Williams G, Najman JM, Al Mamun A. Parent and adolescent reports in assessing adolescent sleep problems: results from a large population study. *Acta Paediatr*. 2016;105(9):e433-e439. <https://doi.org/10.1111/apa.13404>. [↑](#footnote-ref-24)
25. . Landis JR, Koch GG. The measurement of observer agreement for categorical data. *Biometrics*. 1977;33(1):159-174. <https://doi.org/10.2307/2529310>. [↑](#footnote-ref-25)
26. . White SW, Eastwood PR, Straker LM, et al. The Raine study had no evidence of significant perinatal selection bias after two decades of follow up: a longitudinal pregnancy cohort study. *BMC Pregnancy Childbirth*. 2017;17:207. <https://doi.org/10.1186/s12884-017-1391-8>. [↑](#footnote-ref-26)
27. . Houseman EA, Accomando WP, Koestler DC, et al. DNA methylation arrays as surrogate measures of cell mixture distribution. *BMC Bioinform*. 2012;13:86. <https://doi.org/10.1186/1471-2105-13-86>. [↑](#footnote-ref-27)
28. . Heredia FP, Garaulet M, Gómez-Martínez S, et al. Self-reported sleep duration, white blood cell counts and cytokine profiles in European adolescents: the HELENA study. *Sleep Med*. 2014;15(10):1251-1258. <https://doi.org/10.1016/j.sleep.2014.04.010>. [↑](#footnote-ref-28)
29. . Avlund K, Hokland M, Mehlsen MY, et al. Differential associations between white blood cell counts and fatigue in young and older adults. *Aging Clin Exp Res*. 2013;24:439-447. <https://doi.org/10.3275/8473>. [↑](#footnote-ref-29)
30. . Wu Q, Liu JH, Ma QH, Xu Y, Pan CW. White blood cell count as a mediator of the relationship between depressive symptoms and all-cause mortality: A community-based cohort study. *Arch Gerontol Geriatr*. 2021;104343. <https://doi.org/10.1016/j.archger.2021.104343>. [↑](#footnote-ref-30)
31. . Kobrosly R, Wijngaarden E. Associations between immunologic, inflammatory, and oxidative stress markers with severity of depressive symptoms: An analysis of the 2005–2006 National Health and Nutrition Examination Survey. *Neurotoxicology*. 2010;31(1):126-133. <https://doi.org/10.1016/j.neuro.2009.10.005>. [↑](#footnote-ref-31)
32. . Shafiee M, Tayefi M, Hassanian SM, et al. Depression and anxiety symptoms are associated with white blood cell count and red cell distribution width: A sex-stratified analysis in a population-based study. *Psychoneuroendocrinology*. 2017;84:101-108. <https://doi.org/10.1016/j.psyneuen.2017.06.021>. [↑](#footnote-ref-32)
33. . Meng G, Wang L, Wang X, et al. Association between neutrophil to lymphocyte ratio and depressive symptoms among Chinese adults: A population study from the TCLSIH cohort study. *Psychoneuroendocrinology*. 2019;103:76-82. <https://doi.org/10.1016/j.psyneuen.2019.01.007>. [↑](#footnote-ref-33)
